# Editorial: When the Body Feels Like Mine: Constructing and Deconstructing the Sense of Body Ownership Through the Lifespan

**DOI:** 10.3389/fnhum.2022.854135

**Published:** 2022-03-23

**Authors:** Laura Crucianelli, Carissa J. Cascio, Roy Salomon, Gerardo Salvato

**Affiliations:** ^1^Department of Neuroscience, Karolinska Institutet, Stockholm, Sweden; ^2^Department of Psychiatry and Behavioral Sciences, Vanderbilt University Medical Center, Nashville, IN, United States; ^3^Gonda Brain Research Center, Bar-Ilan University, Ramat Gan, Israel; ^4^Department of Brain and Behavioral Sciences, University of Pavia, Pavia, Italy; ^5^Cognitive Neuropsychology Centre, ASST Grande Ospedale Metropolitano Niguarda, Milan, Italy; ^6^NeuroMi, Milan Center for Neuroscience, Milan, Italy

**Keywords:** body ownership, multisensory integration, self-awareness, body representation, lifespan

Bodily self-awareness is a multidimensional construct defined as the feeling that conscious experiences are bound to the self as a unitary entity (Berlucchi and Aglioti, 2010; Blanke et al., 2015; Salvato et al., 2020). A fundamental aspect of bodily self-awareness is the sense of body ownership, described as the awareness of one's body as belonging to oneself and the feeling that a given body part belongs to one's own body (Tsakiris, 2010; de Vignemont, 2011). Even though we all have a body and we usually do not question its very existence, the way in which we build and maintain a coherent sense of body ownership is not fully understood. The last two decades have seen an exponential increase in trying to elucidate its underpinning mechanisms and important studies have significantly advanced the field (see Ehrsson, 2020 for a review). For example, it has been proposed that the integration of exteroceptive, interoceptive, and proprioceptive signals may play a fundamental role in giving rise to the feeling that our body belongs to ourselves (e.g., Park et al., 2016; Crucianelli et al., 2018; Salvato et al., 2020). Nevertheless, several questions remain to be answered. The present Research Topic aimed to better characterize how a coherent sense of body ownership emerges, changes, it is maintained and/or updated throughout the life span and in the case of disorders of body ownership. As such, our Research Topic provides a state-of-the-art overview of the current investigations and topics on the sense of body ownership. It combines interdisciplinary findings from experimental and developmental psychology, neuropsychology, neurology, and cognitive neuroscience, and covers the relation between the sense of body ownership, body awareness, and various cognitive functions in the motor and social domain. We welcomed submissions on the topic ranging from birth to aging, in healthy and pathological conditions, from behavioral, neurophysiological, neuroimaging, and philosophical points of view, as well as more recent virtual reality and technology-oriented research on body ownership.

## Novel Experimental Set-ups to Investigate Body Ownership

In healthy populations, body ownership is mainly assessed and manipulated by means of multisensory illusion paradigms (mainly visual-tactile), which allow to temporary alter the feeling of ownership over a body part or the entire body (e.g., *Rubber Hand Illusion*, Botvinick and Cohen, 1998; Tsakiris and Haggard, 2005, *Full Body Illusion*, Ehrsson, 2007; Lenggenhager et al., 2007, and its *virtual reality* equivalent, Slater, 2009). In this context, a few articles in the present collection used modified versions of these classical bodily illusion methods, which we believe could path the way for future studies aiming at further characterizing body ownership. de Silva et al. provided new evidence on the efficacy of a modified version of the Rubber Hand Illusion (RHI) paradigm (Botvinick and Cohen, 1998), namely the Parasagittal-Mirror-RHI paradigm. This proof-of-concept study combines the use of a parasagittal mirror and synchronous stroking of both a prosthetic hand (viewed in the mirror) and the participant's hand, with a manipulation of the distance between the hands. de Silva et al. showed that the Parasagittal-Mirror-RHI was successful in inducing the illusion of body ownership and the strength of the experience was closely linked to the illusory distance between the rubber hand (reflected in the mirror) and the participant's own hand rather than the actual distance between the two. The result of this study provides important insight into the role of spatial distance of the hands in the way we recognize a body part as our own.

The work by Crivelli et al. offered another novel way to investigate body part ownership by means of the Implicit Association Test (IAT, Greenwald et al., 2003). Participants were asked to complete an IAT of the dominant (vs. non-dominant) hand to the self. There was a linear correlation between the strength of the implicit association of the dominant hand with the self, and such effect increased as a function of hand preference. By implication, this study suggests that the illusion of body ownership might be more effective if applied on the non-dominant hand, toward which healthy individuals have a weaker feeling of ownership. Indeed, their results provided insight into the magnitude of the sense of ownership for one of the two hands, which varies according to the use that the subject makes of the hand in everyday life. The stronger ownership toward the dominant hand could be linked to the fact that such hand plays a more crucial role in motor behavior, and it might interact more extensively with the environment.

## Behavioral, Cognitive, and Affective Consequences of Body Ownership's Manipulations

A growing body of evidence has shown that manipulating the sense of body ownership by means of visuo-tactile paradigms can also induce specific behavioral and physiological changes, such as thermoregulatory and somatosensory processes (e.g., Salomon et al., 2013; Romano et al., 2014; Ricci et al., 2019; Crivelli et al., 2021). Along this line, Ricci et al. showed that transient manipulations of the sense of ownership may alter tactile awareness. During the experiment, healthy participants had to complete a Tactile Quadrant Stimulation (TQS) test while they were exposed to the mirror box, whereby their right hand was reflected and the left one was hidden from view. Results showed that participants reported phantom touch sensation on the hidden left hand, an effect that had previously been observed in patients following stroke. Thus, this study further corroborates the idea that the sense of body ownership can modulate tactile perception, and it provides novel knowledge on the uni- and bilateral representations of touch.

Another study pushed this idea a step further by showing that behavioral changes following manipulations of body ownership might be more profound than previously thought. Clausen et al. demonstrated how body ownership manipulation using an illusory auditive paradigm (*Footsteps Illusion*, Tajadura-Jiménez et al., 2015) may give rise to changes in implicit self-gender associations and explicit self-gender group identification. Across two experiments, Clausen et al. manipulated participants' footstep sounds in real time to resemble more feminine or masculine footsteps during walking. They tested how these sounds changed participants' self-concept and the relation to social groups for cisgender females and cisgender males. Their results showed that females felt more feminine and closer to the group of women after walking with feminine sounding footsteps. Similarly, males felt more feminine after walking with feminine sounding footsteps and associated themselves relatively stronger with the “female” attribute. Thus, auditory-induced body illusions can temporally alter gender identity as well as self-concept and social group identification.

In another study, Burin and Kawashima conducted a randomized controlled trial exposing healthy older participants to illusory sense of body ownership and agency over a moving virtual body. Participants completed two virtual reality high-intensity intermittent exercise sessions, either in a first- or third-person perspective, and they completed cognitive tasks before, in between, and after these two experimental sessions. The results showed that participants observing a virtual body in a first-person perspective performing 20 min of virtual high-intensity intermittent exercise improved their executive functions, and an increase in prefrontal cortex activity was observed following the intervention, as compared to participants performing the sessions in a third-person perspective. As such, this study corroborates the impact of the virtual full-body illusion and its physiological consequences on the elderly, and they further suggest that a longer exposure to those illusions might be necessary to observe significant improvement in cognitive performance.

## Developmental Studies on Body Ownership and Multisensory Integration

The ability to recognize our body as our own arises from complex multisensory integration processes (Blanke, 2012; Ehrsson, 2020), which have been shown to emerge in the early stages of human development (e.g., Filippetti et al., 2013). Along this line, Ratcliffe et al. investigated the relative contributions of visual and proprioceptive inputs on the development of body localization in primary school-aged children. A mediated reality device called MIRAGE was used to explore how the brain weighs visual and proprioceptive information in a hand localization task, whereby children were asked to estimate the position of their index finger after viewing congruent or incongruent visuo-proprioceptive information regarding hand position. Younger children were more accurate in the hand localization task as compared to older children, suggesting that they relied more on proprioceptive inputs and less on visual information. Thus, the results demonstrate that the integration between different sensory inputs starts early in infancy and it optimizes through development, with the bias toward visual information increasing with age.

The contribution to the present Research Topic by Della Longa et al. specifically focused on the difference between pre-term and full-term children in the development of body ownership. The authors investigated whether the deprivation of parent-infant bodily contact in the neonatal period, such as in the case of preterm birth, bears long-term negative consequences for the development of bodily self-awareness. Children completed a RHI, while having EEG continuously recorded, and they performed a pre and post pointing task as well as filling in a questionnaire. Della Longa et al. showed that preterm children present less susceptibility to the RHI, as compared to full-term children, suggesting an atypical integration of multisensory bodily signals. Thus, this study provides an important insight into our understanding of the emergence of bodily self-awareness in pre-term and full-term children, and it corroborates the idea that tactile contact in the first stages of life might play a crucial contribution to the development of a healthy sense of self (e.g., Cascio et al., 2019; Crucianelli and Filippetti, 2020).

## Body Ownership and the Motor System

Previous research indicated that the sense of body ownership is also linked to the motor aspect of the self (for a review, see Seghezzi et al., 2019), as highlighted in the review paper by Liesner et al. The Authors integrated evidence from perception-action interactions, multisensory integration, and developmental psychology to discuss how the sense of body ownership is flexibly updated throughout lifespan. Specifically, a description and mechanistic explanation of “active ownership” is provided, i.e., how humans construct a sense of ownership over the effects of their actions. Liesner et al. suggested that the overlap (or conflict) of interoceptive and exteroceptive sensations is the key factor shaping both active and passive body ownership, and they call for future, more integrative research, encompassing the fields of ideomotor action control, perception and action, and the crucial importance of comparing children in different age groups.

Yizhar et al. used a virtual reality environment to investigate the relationship between sensory and motor cues within a RHI paradigm. Across two experiments, participants viewed their hands switched and mirrored, so that when they moved their hand, they would see the incongruent virtual hand moving. Despite this, participants reported strong body ownership sensation over the virtual hands and the perceived level of agency over hand movement mediated the anatomical congruency effect. Yizhar et al. demonstrated that goal direct agency override plausibility constraints during the RHI paradigm, thus challenging early findings on the importance of the canonical position of the rubber hand during the visual-tactile illusion.

De Coster et al. offers a yet new perspective on the relationship between body ownership and action. This study investigated own-perceived body matching in a more ecological manner as compared to previous studies, namely by focusing on body movement dynamics and clothing cues. Participants were asked to match their own body with a 3D-generated avatar, which was manipulated based on movement dynamics, body size, and fitted clothes. De Coster et al. showed that the accuracy in self-recognition is not significantly influenced by movement dynamics nor fitted clothes. However, confidence about dress fit was higher for dynamic avatars. These findings provide insight for research exploring (own-) body perception and bodily self-awareness and can have implications for future clinical studies with populations characterized by disorders of body representation, such as anorexia nervosa and body dysmorphic disorder.

## Disorders of Body Ownership

We believe that our Research Topic provides some insight into the underlying mechanisms of disorders of body ownership, which may be present also in the absence of a brain lesion. This is the case of Body Integrity Dysphoria (BID), a poorly understood neuropsychiatric disease (Sedda, 2011; Brugger et al., 2013), associated with a persistent urge to amputate one of their healthy limbs. Individuals with BID manifest a puzzling behavioral dissociation. They describe a profound feeling of limb disownership, while they rationally acknowledge the physical presence and biological ownership of body parts (Romano et al., 2015; Saetta et al., 2020; Gandola et al., 2021; Salvato et al., 2022). Addressing this topic, Chakraborty et al. reviewed and discussed current treatment options available for BID, which have proven largely ineffective. Thus, they suggested a novel approach to target and potentially treat people with BID using Brain-Computer Interface (BCI) and neurofeedback. In their mini-review, Chakraborty et al. provided some practical approaches to implicitly promote re-ownership of the limb and engender more positive associations to body representation using BCI, which can target altered patters of brain activity without impairing the anatomical structure and functionality of the individual. This paper is particularly timely in highlighting the urgent need for more effective form of treatment for BID, a clinical condition that can lead to significant distress and life-long suffering.

## Conclusion and Closing Remarks

In conclusion, the evidence produced by this collection of papers provides new knowledge on the way we build, update, and maintain a coherent sense of ownership throughout the lifespan, which is a crucial aspect of our physical and mental wellbeing. As such, it is now clear that to achieve a better understanding of the complexity of the topic of body ownership, we must embrace a multidisciplinary approach. All the contributions to the present Research Topic touched upon different and equally important topics, ranging from perception and action (e.g., touch, sense of agency, and movement dynamics), social cognition (e.g., gender identity and group identification), to developmental and aging psychology, using behavioral, virtual, and neuroimaging methods.

The ample breadth of the contributions allowed this issue to target the multiple dimensions of body ownership. On the one hand, some studies have investigated the factors that contribute to the feeling of recognizing our body as our own; on the other hand, other studies have discussed the behavioral, cognitive, and social aspects that are influenced when manipulating the sense of body ownership. Taken together, we believe that the work here presented in the form of both empirical papers and reviews significantly advances the field of research in body ownership and can stimulate further debate and future research to achieve an even better understanding of how our brain constructs the sense of self and makes sense of the reality around us.

Moving forward, research priorities in this fascinating field are numerous and include, for example, considering a method to assess the sense of body ownership in healthy participants at baseline, without inducing body ownership illusions. Another critical issue to address is understanding the role of different physiological components in the emergence of the sense of ownership, such as respiration, heartbeat, and thermoregulation. Finally, we also believe that this field of research should prioritize the study of pathological ownership in brain-damaged patients; such neuropsychological approach will allow us to build and eventually test theoretical models and infer neuroscientific principles on the construction of the sense of the self. This is important also for developing novel treatments for disorders of body representation, which could apply some of the methods here discussed.

## Author Contributions

All authors listed have made a substantial, direct, and intellectual contribution to the work and approved it for publication.

## Funding

LC was supported by the Marie Skłodowska-Curie Intra-European Individual Fellowship (891175). RS was supported by an Israeli Science Foundation Grant (1169/17). CC was supported by R01MH 102272.

## Conflict of Interest

The authors declare that the research was conducted in the absence of any commercial or financial relationships that could be construed as a potential conflict of interest.

## Publisher's Note

All claims expressed in this article are solely those of the authors and do not necessarily represent those of their affiliated organizations, or those of the publisher, the editors and the reviewers. Any product that may be evaluated in this article, or claim that may be made by its manufacturer, is not guaranteed or endorsed by the publisher.
